# Egg Quality of Italian Local Chicken Breeds: I. Yield Performance and Physical Characteristics

**DOI:** 10.3390/ani13010148

**Published:** 2022-12-30

**Authors:** Chiara Rizzi, Filippo Cendron, Mauro Penasa, Martino Cassandro

**Affiliations:** 1 Department of Agronomy, Food, Natural Resources, Animals and Environment (DAFNAE), University of Padova, Viale dell’Università 16, 35020 Legnaro, Italy; 2 Federazione delle Associazioni Nazionali di Razza e Specie, Via XXIV Maggio 43, 00187 Roma, Italy

**Keywords:** hen, local breed, egg production, egg quality, physical trait

## Abstract

**Simple Summary:**

Biodiversity is one of the key principles of sustainability for food and agriculture. The Veneto region, in Northern Italy, is one of the regions with the highest number of local chicken breeds. Padovana and Polverara are tufted, white eggshell breeds, and different plumage varieties exist. Pepoi, Ermellinata di Rovigo, Robusta Maculata, and Robusta Lionata are dual-purpose and tinted eggshell breeds. The laying rate changed according to the breed. The eggs produced by these breeds differed in weight, eggshell traits (e.g., colour, shape, and thickness), yolk-to-albumen ratio, Haugh units, and meat and blood spots. The consumer can choose a diversified egg quality according to their cooking purposes.

**Abstract:**

The aim of this study was to compare yield performance (from 39 to 50 weeks of age) and egg physical characteristics (at 50 weeks of age) of eight autochthonous chicken breeds of the Veneto region (Italy). Four white eggshell breeds, namely Padovana Camosciata (PA-C, chamois plumage), Padovana Dorata (PA-G, golden plumage), Polverara Bianca (PO-W, white plumage), and Polverara Nera (PO-B, black plumage), and four tinted eggshell breeds, namely Pepoi (PP), Ermellinata di Rovigo (ER), Robusta Maculata (RM), and Robusta Lionata (RL) from a conservation centre were considered in the trial. Significant differences (*p* < 0.05) among breeds were observed for yield performance and egg quality. From 39 to 50 weeks of age, the hen-day egg production was higher in PA-C and RM than in RL, and PO-W and ER were intermediate; PA-G, PO-B, and PP were the lowest. The hen-day egg production changed according to the age of the hens. From 39 to 42 weeks of age, ER showed the highest hen-day egg production and PA-G the lowest; from 47 to 50 weeks, PA-C, PO-W, and RM were the highest and PP the lowest. The tinted eggshell breeds, with the exception of PP, had higher egg weights than white eggshell breeds. PP egg weight was similar to PO-B. As regards the tinted eggshell breeds, RM eggs had the highest eggshell a* and b*, and PP the lowest. PA-C had the most spherical eggs, and PO-B and ER had the most ovoid eggs. PO-W and RM had the highest eggshell thickness and ER had the lowest. The highest eggshell ratio was observed for PO-W and PO-B, and the lowest for ER. The yolk-to-albumen ratio was higher in the white eggshell breeds than in PP, ER, and RL. ER had the highest Haugh units and PA-G the lowest. PO-W, PO-B, PA-C, PA-G, and ER had the lowest egg inclusions, and RL and RM the highest. Tinted eggshell eggs differed from white eggshell eggs by having higher meat spots. Results indicated that the eggs produced by the eight local chicken breeds differed according to the laying rate and a wide range of physical external and internal characteristics which allow the consumer to distinguish them for their genetic origin by the eggshell shape and colour, and to use them for different purposes to valorise poultry biodiversity.

## 1. Introduction

Egg production for human consumption has deeply changed throughout the last century, from small retail points until 1950 to big markets, from eggs laid by hens belonging to local purebreds to eggs laid by hybrid hens [1,2]. Hybrids have been selected for high egg production and their eggs mainly differ in egg weight and eggshell colour [3,4,5], and are consumed as table eggs or egg products worldwide [2,6]. In the last decades, the intensive egg production systems, as well as the health status of the hybrid hens, are two important ethical concerns [7]. A third relevant aspect involved with intensive egg production is biodiversity. Currently, egg production is sustained by hybrids. Recently, there has been a worldwide focus on farming sustainability, including biodiversity [8]. Local breeds and production processes are key points in these goals. Consumers are progressively interested in ancestral food systems [9]. Local breeds represent the historical traditions of a territory, and they show the potential of adapting to emerging conditions including climate change and egg and meat production [8].

The Veneto region, in Northern Italy, has a tradition for poultry production, given its position in the Po Valley, and it has historically produced a high quantity of maize and cereals, which are the main ingredients of the chicken diet. At the beginning of the last century, several breeds were reared in the farms of the region, but they were gradually lost to the increasingly intensive production systems based on hybrids [10]. Fortunately, some breeds still exist and are under conservation plans. Some of the Veneto chicken breeds have a very old history, such as Padovana, cited by Aldrovandi since 1600 [11]. This breed probably came to Italy in the XIV century, having been brought from Poland [12]. Polverara, which originated from Padovana, is a traditional breed of the Veneto region as well as Pepoi [13]. In the middle of the last century, three breeds were created in the Veneto region [13]. Currently, conservation programs for these breeds have been carried out by poultry conservation centres in the Veneto region, and previous research has provided data on the growth, meat production [14,15,16,17], and egg traits [18,19,20,21] of these local breeds. Moreover, genetic characterization of 23 Italian chicken breeds, including those presented in the current study, has been carried out [22,23].

The aim of this study is to compare the egg production and egg physical characteristics of eight local chicken breeds of the Veneto region.

## 2. Materials and Methods

### 2.1. Hens and Rearing Conditions

The hens included in the trial belonged to breeds of the Veneto region, Northern Italy. Padovana (PA) and Polverara (PO) breeds produce eggs with white eggshells, and two plumage varieties for each breed were studied: Padovana Camosciata (PA-C, chamois plumage), Padovana Dorata (PA-G, golden plumage), Polverara Bianca (PO-W, white plumage), and Polverara Nera (PO-B, black plumage). Pepoi (PP), Ermellinata di Rovigo (ER), Robusta Maculata (RM) and Robusta Lionata (RL) lay eggs with tinted eggshells. The origins of these breeds and the main features of the hens are summarized in Table 1. The birds show a slow-growing rate as they reach adult body weight after 6–7 months of age [13,14,15,16,17]. PA and PO are two old breeds selected for different plumage colours and with good laying activity [13]. The adult body weight of PO and PA ranges from 1.3 to 2.1 kg. PP is an old local breed of small body size (average adult body weight = 1.2 kg), with good egg production and discrete breast muscle development [13]. The dual-purpose (meat and egg) medium-body size breeds were created in the Veneto region (Italy) during the 1950s, using Sussex and Rhode Island (ER breed), and Brown Orpington and White America (RM and RL breeds) [13]. The adult body weight ranges from 2.2 to 2.6 kg for ER and 2.8 to 3.3 kg for RM and RL [13].

Sixty birds per breed were reared under a free-range farming system from 8 weeks of age when the outdoor temperatures and relative humidity allowed the birds conditions promoting their wellbeing. The chicks hatched during the same period, in spring; during the first 4 weeks of life, the birds were kept indoors on litter, under infrared radiation lamps, at an environmental temperature decreasing from 32 to 24 °C. After 8 weeks in indoor conditions, the birds had free access to outdoor spaces. Each breed had access to indoor (4 hens/m^2^) and outdoor (5 m^2^/hen) spaces, divided by netting. In the outdoor space of each breed, the grass growth was not high, due to the previous absence of rain throughout the summer months and the prolonged walking activity of the birds throughout the next months, which did not allow for high regrowth. In the indoor spaces, the floor was covered by wood shavings. On the floor, wooden nests and perches were available to the hens. The birds were subjected to the same prophylaxis procedures, rearing conditions (temperature, photoperiod), and feeding from the time of hatching through the laying period. The animals were fed ad libitum different feeds: one throughout the growing period until the pubertal age, and one throughout the laying period (39 to 50 weeks of age) based on maize and soybean (average composition, as fed basis: crude protein = 16.2%, Ca = 4.2%, P = 0.6%, lysine = 0.7%, methionine = 0.3%, and metabolizable energy = 11.5 MJ/kg, without any pigment addition for yolk colour). The pubertal phase and the beginning of laying activity were at the end of summer through the beginning of autumn for PP, ER, RM, and RL, when the photoperiod and environmental temperature, at the latitude (45°02′53′’ N) of the Veneto region, were decreasing. After about 2.5 months of laying activity, their egg production stopped. From December to the end of the trial (March), the natural photoperiod was complimented by artificial light inside the rooms, where the hens spent the night, to obtain a photoperiod of 14L:10D. The PA and PO breeds started laying after the first weeks of artificially complimented photoperiod. The trial started in January (at 39 weeks of age) when the hens of all the breeds were in laying activity and lasted in March (at 50 weeks of age). The environmental temperature varied from 5 °C to 12 °C.

### 2.2. Yield Traits

The eggs were collected according to European Regulations (EC No. 1/2005 and EC No. 1099/2009) on animal care and welfare. The sampling did not affect the welfare of the hens as it was carried out when the animals were not in the nests, thus avoiding their handling. The hen-day egg production (number of eggs/number of live hens × 100) was checked daily from January, when the hens were 39 weeks old, until March. At 49–50 weeks of age, throughout two weeks, the mean daily weight of the eggs (average based on the total daily eggs considering samples of approximately 20 eggs per breed) were checked for each breed. The hen-day egg mass was calculated as hen-day egg production (%) × daily egg weight (g).

### 2.3. Physical Analyses of the Eggs

At 50 weeks of age, when all the breeds had been laying since about 12 weeks, on each daily collection of about 15 eggs, carried out throughout 4 days, the eggshell and internal traits were analysed, excluding the defective eggs (double yolk, abnormal shell). Given the hen-day egg production of the studied breeds and the number of hens per breed, the daily egg collection allowed a representative number of eggs for each breed for further analyses. All the measurements were carried out on a one-day old egg. On each egg, external and internal traits were measured to obtain an adequate number of observations per breed: external eggshell traits and weight of every single egg (60 observations: 15 eggs/day × 4 days), eggshell thickness (35 observations: 8–9 eggs/day × 4 days), egg components (25 observations: 6–7 eggs/day × 4 days), Haugh units (22 observations: 7–8 eggs/day × 3 days), yolk colour (9 observations: 4–5 eggs/day × 2 days), meat and blood spots (60 observations: 15 eggs/day × 4 days). Each egg was weighed, and the eggshell length (along the longitudinal axis) and width (along the equatorial axis) were measured by callipers (0.01 mm). The eggshell colour was measured by a colorimeter Chroma meter CR 300 (Minolta Co Ltd., Osaka, Japan) using the CIE scale [25]: L, a* and b* reflect lightness (0 = black, 100 = white), redness (−100 = green, 100 = red), and yellowness (−100 = blue, 100 = yellow), respectively. The shape index (%) was calculated as the ratio between the width and the length of the egg (width/length × 100). The egg surface area (cm^2^) and volume (cm^3^) were calculated as [26]:

Surface area = (0.9658 × W/L + 2.1378) × L × W

Volume = 0.525 × L × W^2^

where W = width (cm) and L = length (cm).

The eggshell thickness was measured by means of a digital calliper (0.001 mm) (Mitutoyo, Japan) at the equatorial level of each egg. The yolk was manually separated from the albumen three times and weighed, and the albumen weight was calculated as the difference between the egg weight and the weight of the yolk and eggshell. Before weighing the eggshell, it was cleaned from residues of albumen and then dried for 12 h at 45 °C. The eggshell ratio (%), albumen ratio (%), and yolk ratio (%) were calculated as the ratio between each egg component and egg weight × 100. To evaluate the physical internal quality of albumen and yolk, each egg was broken, and the yolk and albumen were put on a glass plate to detect the inclusions (blood and meat spots) by means of a mirror placed under the glass. For measuring the albumen height, a micrometer (0.01 mm) (Mitutoyo Co, Kawasaki, Japan) was used. The yolk colour was measured by the DSM yolk fan (formerly Roche scale and indicated as Roche colour). Egg weight and albumen height were used to calculate the Haugh Units (HU) as measured by Haugh (1937) [27].

### 2.4. Statistical Analyses

The effect of breed on the hen-day egg production was evaluated from 39 to 50 weeks of age and at three ages (39 to 42 weeks, 43 to 46 weeks, and 47 to 50 weeks) using a factorial model 8 × 3. The data on the hen-day egg mass (49–50 weeks) and egg physical traits (50 weeks) were analysed by one-way ANOVA, considering the breed effect. The GLM procedure of SAS (SAS Institute Inc., Cary, NC, USA) was used for this purpose. Tukey’s post-hoc test was performed for testing statistical differences between the groups. For testing statistical differences for blood and meat spots, the chi-square test was used. Given that in the PA-C and PA-G, and the PO-W and PO-B, the meat and blood inclusions were very low, PA-C and PA-G, and PO-W and PO-B were considered as two breeds (PA and PO, respectively) when testing differences for meat and blood inclusions. Significance was set at *p* < 0.05.

## 3. Results

### 3.1. Yield Performance

**Figure 1:**
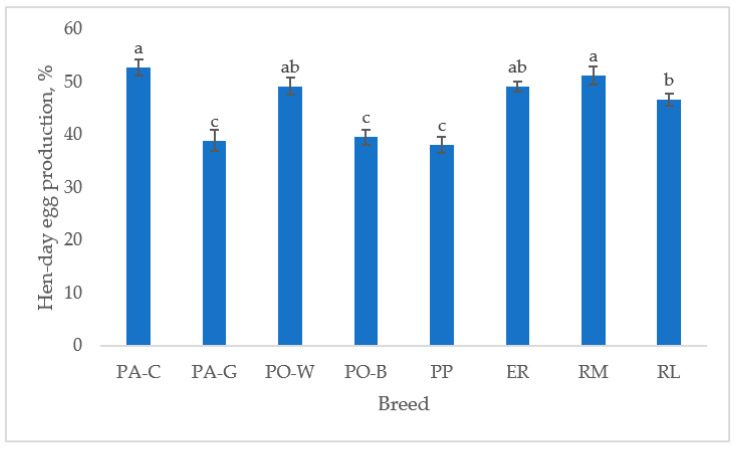
Least squares means (with SE) of hen-day egg production (from 39 to 50 weeks of age; observations (*n*) = 85 per breed). PA-C: Padovana Camosciata; PA-G: Padovana Dorata; PO-W: Polverara Bianca; PO-B: Polverara Nera; PP: Pepoi; ER: Ermellinata di Rovigo; RM: Robusta Maculata; RL: Robusta Lionata. Different letters indicate statistically different means (a,b,c: *p* < 0.05).

The hen-day egg production (Figure 1), from 39 to 50 weeks of age, differed between breeds (*p* < 0.05). For the white eggshell hens, PA-C was higher than PA-G and PO-W was higher than PO-B (*p* < 0.05). For the tinted eggshell breeds, the hen-day egg production was higher in RM than in RL (*p* < 0.05), ER was intermediate, and PP was the lowest (*p* < 0.05). PA-C and RM showed the highest hen-day egg production, and PA-G, PO-B, and PP had lower egg production than the other breeds (*p* < 0.05).

**Figure 2:**
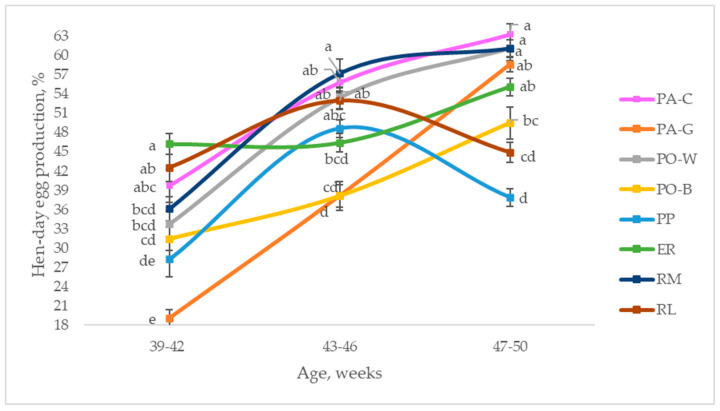
Least squares means (with SE) of hen-day egg production according to the age of the hens (from 39 to 42 weeks; from 43 to 46 weeks; from 47 to 50 weeks; observations (*n*) = 28 per period and breed). PA-C: Padovana Camosciata; PA-G: Padovana Dorata; PO-W: Polverara Bianca; PO-B: Polverara Nera; PP: Pepoi; ER: Ermellinata di Rovigo; RM: Robusta Maculata; RL: Robusta Lionata. Different letters between breeds within age indicate statistically different means (a,b,c,d,e: *p* < 0.05).

Figure 2 depicts the hen-day egg production of the eight breeds throughout the studied period, from 39 to 50 weeks of age, comparing the laying rate of the breeds at three ages. In the first period (39 to 42 weeks of age), ER was higher than RM, PO-W, PO-B, PP, and PA-G (*p* < 0.05), and the other breeds were intermediate. PP and PA-G were the lowest (*p* < 0.05). From 43 to 46 weeks of age, the laying rate increased for all the breeds; extreme values were observed for RM and PA-G, as they showed the highest and the lowest laying rates, respectively (*p* < 0.05). PA-C, PO-W, and RL reached higher values than those of PA-G and PO-B (*p* < 0.05), whereas ER and PP were intermediate. Throughout the third period (47 to 50 weeks of age), the hens had divergent trends, according to the breeds. Most of them (PA-C, PA-G, PO-W, PO-B, ER, RM) increased the laying rate, even if with a tendential trend for some of them, whereas PP and RL decreased it. The hen-day egg productions of PA-C, RM, and PO-W were higher than those of PO-B and RL (*p* < 0.05), whereas PA-G and ER were intermediate; PP showed the lowest laying rate (*p* < 0.05).

**Figure 3:**
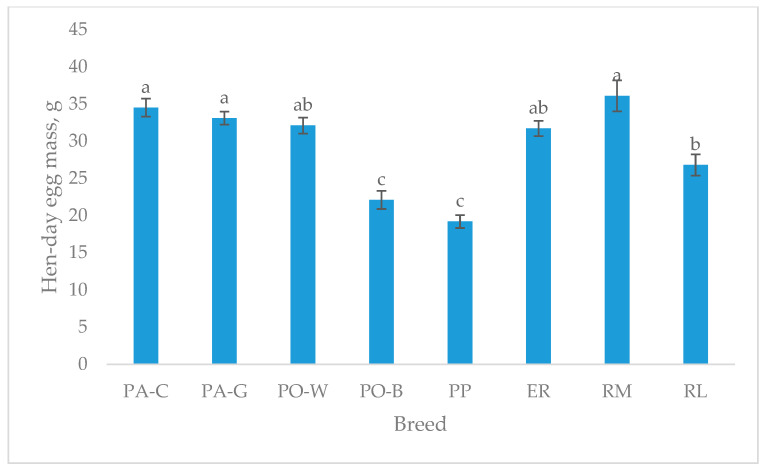
Least squares means (with SE) of hen-day egg mass (from 49 to 50 weeks of age; observations (*n*) = 14 per breed). PA-C: Padovana Camosciata; PA-G: Padovana Dorata; PO-W: Polverara Bianca; PO-B: Polverara Nera; PP: Pepoi; ER: Ermellinata di Rovigo; RM: Robusta Maculata; RL: Robusta Lionata. Different letters indicate statistically different means (a,b,c: *p* < 0.05).

The hen-day egg mass (Figure 3), which refers to the age of the egg sampling (49–50 weeks), shows higher values for PA-C, PA-G, and RM than RL. PO-W and ER were intermediate, and PO-B and PP were the lowest (*p* < 0.05). For the white-eggshell breeds, PO-B was the lowest, and for the tinted eggshell breeds, the lowest were RL and PP (*p* < 0.05).

### 3.2. Egg Weight

**Figure 4:**
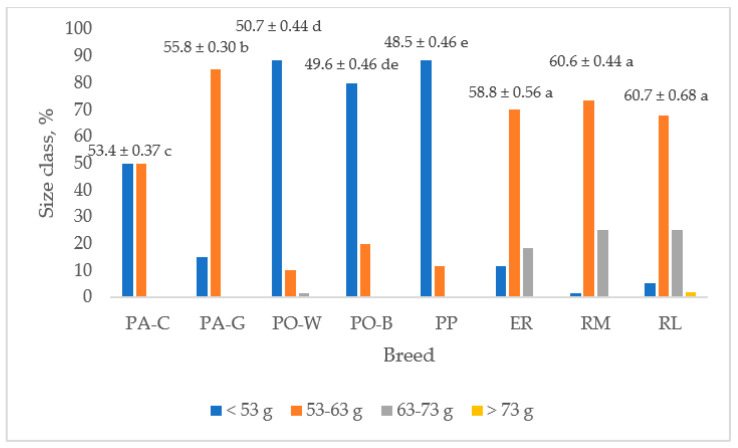
Egg weight (least squares means ± SE) and size class frequency of the breeds (PA-C: Padovana Camosciata; PA-G: Padovana Dorata; PO-W: Polverara Bianca; PO-B: Polverara Nera; PP: Pepoi; ER: Ermellinata di Rovigo; RM: Robusta Maculata; RL: Robusta Lionata). Different letters indicate statistically different means (a,b,c,d,e: *p* < 0.05). Observation (*n*) = 60 per breed.

Figure 4 shows the egg weight and the frequency of the size classes for each breed. The ER, RM, and RL breeds had higher egg weights than that of PP and those of the white eggshell breeds (*p* < 0.05). The PA-G egg weight was higher than that of PA-C (*p* < 0.05), and PO-B and PO-W egg weights were the lowest (*p* < 0.05). PP, followed by PO-B and PO-W, showed the lowest egg weight. As the frequency of the size class is concerned, the ER, RM, and RL hens laid eggs mainly of medium size (70%), followed by large size (23%) and small size (6%). Only RL laid very large size eggs, but at a low level (1.7%). The PP eggs were of small (88%) and medium size (12%).

PA-C showed similar (50%) percentages of small (<53 g) and medium eggs (53–63 g), whereas for the PA-G breed the medium size was prevailing (85%), and a lower percentage was shown for the small size (15%). PO breeds gave a high percentage of small-size eggs (88% in PO-W and 80% in PO-B) and a lower percentage of medium-size eggs (10% in PO-W and 20% in PO-B). Only PO-W laid large-size eggs, but at a very low level (1.7%).

### 3.3. Eggshell Traits

Table 2 shows the eggshell traits of the breeds. The eggs of RM and RL had the highest width, followed by ER, PA-C, and PA-G (*p* < 0.05); PP, PO-W, and PO-B were the lowest (*p* < 0.05). The egg width was higher in PA than in PO eggs (*p* < 0.05). The eggs of ER and RL showed the highest length (*p* < 0.05), followed by PA-G (*p* < 0.05), and those of RM were intermediate. PA-C and PO-B were lower than PA-G (*p* < 0.05), and PP was the lowest (*p* < 0.05). The shape index showed marked differences only between a few breeds: PA-C showed the highest (*p* < 0.05) shape index and PO-B and ER the lowest (*p* < 0.05). The other breeds were intermediate, being similar to PA-C (PP, RM, and PO-W), and PO-B and ER (RL and PA-G).

The area-to-volume ratio was higher in PP, PO-W, and PO-B than in PA-C and PA-G (*p* < 0.05), and ER, RM, and RL were the lowest (*p* < 0.05).

The lightness (L) differed among the white eggshell breeds, being higher in PA-G than in PA-C and PO-B (*p* < 0.05), whereas PO-W was intermediate. All the tinted eggshell breeds showed a lightness lower than that of the white eggshell breeds (*p* < 0.05). They showed different values, as PP was higher than RL, which was higher than ER, and RM was the lowest (*p* < 0.05). The a* index (redness) and the b* index (yellowness) differed among the tinted eggshell breeds, being higher in RM, followed by ER, RL, and PP (*p* < 0.05). The white eggshell breeds did not differ for a* and b* (with the exception of PA-G, which showed the lowest b* value, *p* < 0.05), which were lower than those of the tinted eggshell breeds (*p* < 0.05). The eggshell thickness was similar in RM and PO-W, which were higher than PA-C, and ER was the lowest (*p* < 0.05). The eggshell weight was higher in RM than in PA-C, ER, and PP (*p* < 0.05). The eggshell percentage was higher in PO-W and PO-B than in PA-C, PA-G, and RL (*p* < 0.05); ER was the lowest (*p* < 0.05).

### 3.4. Albumen and Yolk Traits

Table 3 shows the albumen and yolk traits of the eggs laid by the eight breeds. ER, RM, and RL showed higher albumen weights than those of PP and white eggshell breeds (*p* < 0.05). PA-G was higher than PO-B (*p* < 0.05), which was the lowest (*p* < 0.05), and the other breeds were intermediate.

The highest albumen ratio was observed in RL, followed by PP, then RM, PA-C, and PO-W (*p* < 0.05). The lowest was PO-B (*p* < 0.05). ER was intermediate between RL and PP, and PA-G was intermediate between PA-C and PO. ER had the highest Haugh units, followed by PA-C, PO-W, and PO-B, and the lowest values were for PA-G (*p* < 0.05). PP was similar to ER, and RM and RL were more similar to the white eggshell breeds.

Yolk weight showed the highest values in RM and PA-G, and the lowest in PO-W, followed by PP (*p* < 0.05). The PA-G showed the highest yolk ratio, whereas the lowest was in the tinted eggshell breeds (*p* < 0.05), with the exception of RM, which was more similar to the white eggshell breeds. PO-B was intermediate between PA-G and PA-C. Similar results were observed for the yolk-to-albumen ratio. It was higher in PO-B than in PA-C and PO-W (*p* < 0.05). PP, ER, and RL showed the lowest yolk-to-albumen ratio (*p* < 0.05), and RM was similar to PA-C and PO-W.

**Figure 5:**
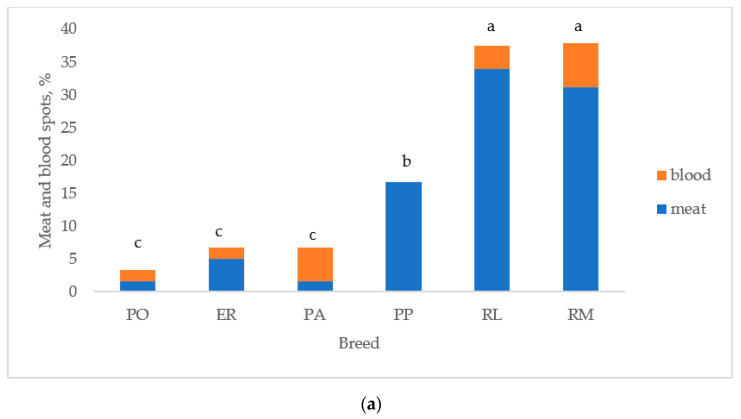

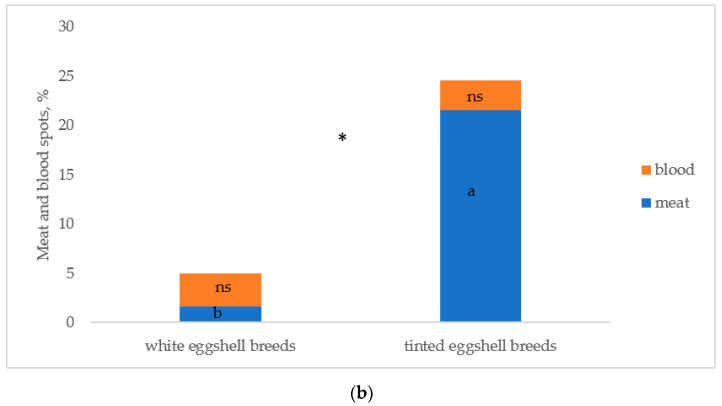
Albumen and yolk inclusions for the (**a**) breeds and (**b**) white and tinted eggshell breeds (PO: Polverara; ER: Ermellinata di Rovigo; PA: Padovana; PP: Pepoi; RL: Robusta Lionata; RM: Robusta Maculata). Different letters between breeds (**a**) and between the same colours of white (PO, PA) and tinted (PP, ER, RM, RL) eggshell breeds (**b**) indicate statistically different values (a,b,c: *p* < 0.05; ns: not significant). Statistical difference between white and tinted eggshell breeds (**b**) is indicated (*: *p* < 0.05). Observations (*n*) = 60 per breed (**a**); observations (*n*) = 240 per eggshell colour group (**b**).

Figure 5 reports the albumen and yolk inclusions of the eggs laid by the breeds. As previously indicated, the white eggshell breeds were considered as two breeds (PA and PO) to test differences between breeds. Figure 5a reports the breeds according to the increasing values of total inclusions: PO, ER, and PA differed (*p* < 0.05) from RL and RM, which were similar. PP was intermediate between the first three breeds (*p* < 0.05) and RL and RM (*p* < 0.05). Total inclusions (Figure 5b) differed (*p* < 0.05) between white and tinted eggshell breeds for a higher (*p* < 0.05) presence of meat spots in the tinted eggshell breeds, whereas blood spots were similar.

**Figure 6:**
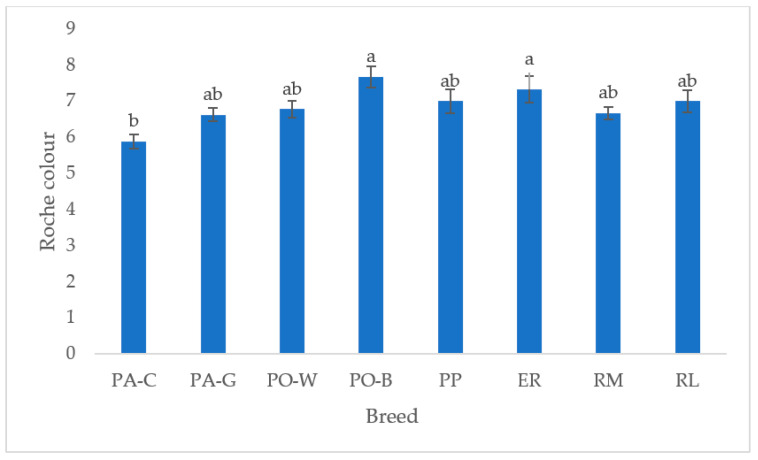
Least squares means (with SE) of yolk Roche colour (PA-C: Padovana Camosciata; PA-G: Padovana Dorata; PO-W: Polverara Bianca; PO-B: Polverara Nera; PP: Pepoi; ER: Ermellinata di Rovigo; RM: Robusta Maculata; RL: Robusta Lionata). Different letters indicate statistically different means (a,b: *p* < 0.05). Observations (*n*) = PA-C (9), PA-G (8), PO-W (9), PO-B (9), PP (9), ER (9), RM (9), RL (7).

In Figure 6, the yolk colour of the white and tinted eggshell breeds is depicted. PO-B and ER were the highest, differing from PA-C (*p* < 0.05), whereas the other breeds were intermediate. The white eggshell breeds were more differentiated than the tinted eggshell breeds.

## 4. Discussion

This trial compared local breeds characterized by different genetic background, despite not well known in some cases. The white eggshell breeds seem to differ from the tinted eggshell breeds due to a different genetic origin based on different species of the wild *Gallus* [28,29]. The mean hen-day egg production of the studied breeds was about 50% for five breeds (PA-C, PO-W, ER, RM, and RL) and about 40% for three breeds (PA-G, PO-B, and PP). These data are not comparable to those of hybrid hens of similar age, selected for their high production [3,4,5]. In a previous trial, hybrid hens, reared under outdoor conditions, showed yield performances lower than those performed under controlled conditions, and higher than those of the purebred hens [20]. Furthermore, the hen-day egg production shown by the ER and RM hens of the current trial was not very different from those shown by the hens belonging to the same breeds and at similar ages, even if checked at a different season [20]. The onset of laying differed among these breeds, as the tinted eggshell breeds started laying at about 31 weeks of age, at the end of summer and beginning of autumn, in the presence of a natural and decreasing photoperiod, whereas the white eggshell breeds started many weeks later, under an increasing and artificially complimented photoperiod. Many are the factors affecting the timing of sexual maturation and the onset of laying in the hen [30]. Adequate development of body size, which also makes the bird photoresponsive to change in photoperiod, the length of photoperiod, the sensitivity to the lightness and photoperiod, and the environmental temperature may be involved in this process. It is worth remembering that in the presence of a good development of the body size of the bird, the hypothalamic-pituitary axis is sufficiently developed for stimulating the release of the hormones involved in reproduction [30]. Furthermore, a day length shorter than 10 h may exert a direct retarding influence on the hypothalamic-pituitary system [30]. The white eggshell breeds have an adult body weight lower than that of brown eggshell breeds (Table 1), given that the first breeds show less muscle development and, thus, they could reach adult body weight earlier, in comparison to the brown eggshell breeds [28]. It is hypothesised that, in the presence of a decreasing photoperiod, lightness, and environmental temperature, the birds characterized by a lower adult body weight, as the white eggshell breeds, responded with a delay of laying activity for preparing their body conditions and satisfying the body thermal regulation requirements during the coldest months. As stated by Ghigi, chicken breeds may be divided into three groups according to their origin and eggshell colour [28]. As far as the phenotype is concerned, white eggshell breeds differ from brown eggshell breeds for smaller body size and higher egg production. Also, metabolism and hormonal profile are involved in their different origin. The PP hens are considered as belonging to a tinted eggshell and dual-purpose breed, but they show a small adult body size, lower than that of the white and the other tinted eggshell breeds, and the onset of laying was similar to these last. Although its genetic position has been found between the white and tinted eggshell breed groups, and especially between PO and ER [23], the origin of this breed is still uncertain. It is worth remembering, also, that the PA and PO are tufted breeds (Table 1) [12,13], with different perceptions and sensitivities to light, which is absorbed by photopigments via the retina of the eye and penetrates bone and cranial tissues [30]. For the crested Polish chicken, from which the PA breed seems to derive, as it is characterized by a cranial hernia, large differences in the brain composition were found when compared to other breeds [31]. Differently, as stated above, all the tinted eggshell breeds, and especially ER, PP, and RM showed an earlier onset of laying than the white eggshell breeds. These aspects need more knowledge, as these breeds are usually reared outdoors, and their responses to environmental factors may differ. Moreover, the management in terms of hatching season for the chicks, as well as the implementation of natural photoperiod with artificial light, may vary according to the farm, thus affecting in different ways the physiological and productive responses of the hens.

Egg size is one of the traits which producers, transformers, and consumers are interested in. The PA, PO, and PP hens produced eggs of small and medium sizes. PO and PP produced mainly (80%) small-size eggs, whereas PA produced medium-size eggs, with differences between the plumages being chamois and golden, this last plumage with a higher value (85% vs. 50%). The ER, RM, and RL hens produced eggs classified into the first three size classes, with about 70% medium-size eggs, followed by large-size eggs (21%) and small-size eggs, especially ER (12%). RL hens gave eggs classified as very large size, even if at a low percentage (1.7%). The results indicate that more than 50% of the egg production of PA-C, PO, and PP are small-size eggs, generally not sold in the retail market, and only PA-G gave more than 80% medium-size eggs.

Currently, egg weight, the rearing system, and special diet are indicated on the carton of table eggs, which the consumer considers in the retail market [32]. Generally, the paper carton contains hybrid eggs of medium, large, and very large size, whereas the small size eggs, laid by hybrid hens at the beginning of the productive cycle, are used for egg products [6]. Previous research stated that table eggs produced by Italian native chicken breeds are sold in niche markets and their price is similar to or higher than that of organic eggs [33].

The egg weight mainly varies according to the genotype (body weight) and the age of the hen, both in hybrids and in purebreds [3,4,5,34,35]. Even if the body weight and the feed intake capacity of the hen are important factors affecting the egg weight, different effects of the body size and oviduct development and activity may occur according to the genotype of the hen. Furthermore, chicken genetics explains only a part of the expressed egg size, as the environment has a relevant effect on overall results and different management and nutritional tools may deeply affect the egg weight [36]. The results of this trial on egg weight confirm this trend, as the eggs produced by the breeds with lower body weight showed lower size (PP, PA, and PO) than those laid by the heavier breeds (ER, RM, and RL). The effect of the body size may be more evident in the purebreds, which show different body sizes according to their plumage colour variety [16,24,37]. In the current study, variations in the egg size classes were observed among breeds. The body weight increases with the hen ageing. At 50 weeks of age, a bird has reached the adult body weight and a complete oviduct development. It is presumable that, likewise in previous research [20], this condition should have been performed by the birds of all the breeds studied which produced eggs of different size classes. In previous trials, the studied breeds showed an increase in egg weight, from 31 to 43 weeks of age, of about 14.0% for both ER and RM [35], and, from 36 to 50 weeks of age, of about 11.0% for both ER and PP [21]. However, the laying activity of these hens reared under outdoor conditions continued throughout the next months, and thus possible changes, beyond 50 weeks of age, in egg production and the percentage of each egg size class, according to each breed, could have occurred.

Egg size, especially for the purebreds, may be considered, also, with other quality traits which could describe an egg quality profile for each breed and address it to different purposes and uses in cooking preparation.

The eggshell traits are important both for the producer and the consumer, as they may affect many egg properties, and, when they are peculiar for the quality profile of each breed, may identify the breed of the egg-laying hen, also.

The eggshell colour is a trait which may largely influence the first choice of the consumer [38], with differences among countries, often based on historical traditions [39].

The pigments found in the shells of domestic hens are protoporphyrin IX, biliverdin, coproporphyrin, and uroporphyrin, and their production depends on many factors [40]. For the eggshell colour, the presence of genetic links [41], as well as several genes that code for proteins and enzymes regulating the production and deposition of pigments, has been indicated [40]. The deposits of the pigments are mainly located within the shell gland, but their production varies for the sites of secretion and time of deposition on the eggshell according to the breeds [40]. Pigment production is affected also by some hormones and some components of the diet. Particularly, the eggshell colour shows a higher variability under free-range conditions than under a controlled environment [42]. For the brown eggshell genotypes, being the colour purely of genetic origin, crosses between white and brown eggshell genotypes result in intermediate colours, due to a codominance effect [40]. Wardecka et al. [43] indicated the presence of a sex-linkage association for the concentrations of the eggshell pigments, as the colour changed according to crosses between males of brown eggshell breeds and females of white eggshell breeds, and to the reverse. In this trial, the eggshell colour of the studied breeds widely differed among the breeds, due to probable effects of all the previously cited factors, however, the exact mechanism is unknown. For the white eggshell breeds, the pigment deposition occurred at different levels with significant differences in lightness among all the breeds, and in the redness and yellowness index between PA-G and the other breeds. For the tinted eggshell breeds, the differences in these colour traits were more marked, as a consequence of the different genetic origins of the four breeds. The tinted eggshell breeds showed different colours, going from a very pale brown (PP) to a moderately deep brown (RM). PP is an old breed, genetically near to the white eggshell breeds [23], and traits such as eggshell colour and egg size seem to confirm this closeness. ER was created using Sussex males and Rhode Island females, and the cross between white and brown eggshell breeds gave birds laying eggs with a medium brown shell. Visually, the ER eggshell shows a colour less uniform than those of RM and RL, as the deposition of the pigments at the level of pores seems to be more marked. This condition may depend on the time and sites of secretion and deposition of the pigments, as they could be more deposited in the eggshell or the cuticle [40,44]. RM and RL showed differences in lightness, redness, and yellowness index. RM showed a more coloured eggshell than RL. These two breeds were created using the same breeds, Brown Orpington and White America, both brown eggshell breeds, but with different crosses, and the hens of the two breeds laid eggs differing for other eggshell traits. The eggshell colour may differ according to other traits, such as egg size and shape. At 50 weeks of age, the egg weight of RM and RL was similar, even if different percentages of each egg size class were checked, and the RM eggshells were more spherical and with a higher thickness than those of RL. As indicated previously, eggshell colour depends on many factors involved, especially eggshell gland characteristics. More knowledge is needed to elucidate the eggshell formation of all these breeds and to clarify the involved genes, the times and the sites of the production, and the deposition of pigments, as well as their kind.

For table eggs, many are the eggshell traits involved with the resistance of the egg and its internal quality during storage [35,44]. The egg shape is important for the producer and the consumer, as a spherical egg is more resistant to external physical insults than an ovoid egg, but also eggshell thickness is a trait involved with eggshell resistance [40]. For the white eggshell breeds, plumages containing black colour gave more ovoid eggs than those of the other plumages. For the tinted eggshell breeds, RM showed the most spherical eggs, especially when compared to those of ER and RL. The shell membranes fix the shape of the eggshell rather than the shell itself [45]. In the oviduct, the magnum-isthmus junction, where membrane deposition is initiated, is the site of shape determination. The final shape, after the isthmus tract, is due to pressure differences across the closed elastic membrane with inhomogeneous properties, owing to variations in thickness and material properties. Initially, on the membrane, surrounding the sphere of yolk and albumen, internal pressure is created inside the membrane-enclosed sac, due to many factors, such as water absorption, differential elasticity of the isthmus wall, muscular contractions, or some combinations of these. Differences in membrane material properties, including collagen fibre composition and direction, elastic modulus, and thickness, induce a differential distortion in response to pressure in the axial and azimuthal direction.

The area-to-volume ratio, which is higher in an ovoid egg, is important during egg storage [46]. An adequate area-to-volume ratio may be a valid barrier for a good passage of gases and water throughout the internal egg environment and the atmosphere. Also, the eggshell thickness and colour may be important. The white eggshell shows a generally higher thickness than the coloured eggshell [47]. This result found also in previous research [20], seems to confirm a genetic component for this trait. Four key genes coding for ion transport were found to contribute significantly to the regulation of the eggshell thickness, besides the expression of the *CALB1* gene, which is associated with calcium transport in the intestine and uterus, and that of the *MP4* gene, which codes for a protein used to bind calcium to the shell [40]. Besides the expression of some genes, the thickness may be affected by other factors involved with egg production and egg size. The lower eggshell thickness showed by PA when compared to PO, may be due to the hen-day egg mass, checked in the last two weeks, which was higher in PA. A decrease in the shell thickness may indicate that the same amount of calcium is distributed over a larger surface area of the shell [44]. In this trial, the PA and PO eggshell thicknesses were higher than those of the coloured eggshell breeds, with the exception of RM, due to their spherical shape and the intensity of brown colour. This result agrees with previous studies which reported that thickness, egg production being equal, may vary according to the egg shape, being higher in spherical eggs, and, for the coloured eggshell, to the pigment content, being higher in deep brown eggshells [40,44]. The shell structure strength was also found to be related to pigmentation [48] and a correlation has been identified with brown-pigmented eggshells [49]. Furthermore, an appropriate eggshell structure and thickness may be a barrier against the entrance of pathogens inside the eggs, even if the control of pathogens throughout the eggshell depends also on the cuticle and the dimensions of pores [44,47]. Given that the eggshells of white egg breeds seem to differ in thickness and structure from those of the tinted egg breeds, the pigment deposition of these last breeds seems to be important for the internal quality of the egg. Pigments, according to their origin, site, and modality of deposition in the eggshell and cuticle, may exert different effects [40].

The eggshell ratio, as a consequence of its dimensions and mineral content, differed between the studied breeds. Any comparison with the results of previous trials is difficult as the changes in the eggshell weight and ratio are affected by the breed, age of the hens and the different environmental conditions studied [35,44,50,51]. Even if, for the consumer, the eggshell is considered a useless component, a good weight and shape must ensure a good eggshell resistance and barrier.

The eggs produced by these local breeds showed differences also in internal quality. Most of the internal egg quality traits showed differences according to eggshell colour, which seems to be the main factor identifying their genetic origin. The quantity of yolk and albumen in each egg depends not only on the egg weight but on the genotype of the hen, as the breeds showed a different ratio between yolk and albumen. In this trial, the white eggshell breeds showed a yolk-to-albumen ratio higher than 0.50, varying from 0.56 to 0.61. The tinted eggshell breeds PP, ER, and RL showed a yolk-to-albumen ratio of about 0.50, and only RM reached 0.56. The eggs of indigenous chicken breeds, without any genetic selection, addressed to changing the weight of the egg components, show a higher yolk-to-albumen ratio than that of other avian species, to supply more nutrients for young birds to survive independently [52]. These data agree with those found in eggs of other purebreds [50,51,53] and differ from those of hybrid hens, which show a yolk-to-albumen ratio less than 0.50 [50,51,52] as a consequence of a selection process addressed to heavier eggs obtained with an increased weight of the albumen and, thus, with a lower lipid content proportion, also.

Yolk and albumen spots, originating, respectively, during ovarian follicle rupture and during the albumen secretion by the oviduct [54], are undesired inclusions for the consumer. The two groups of breeds differed for these traits, as white eggshell breeds showed a percentage of blood and meat spots lower than those of the tinted eggshell breeds. Furthermore, in this last group, the blood and meat spots differed among the breeds, and ER showed lower values when compared to the other breeds, especially for meat spots. These data confirm the results of previous trials [20,53], which found that the eggs laid by white egg breeds show a lower level of yolk and albumen inclusions. When compared to purebred eggs, the eggs of hybrids show a low percentage of inclusions, because of genetic selection [34,55].

The main properties of albumen are addressed to protect the embryo against pathogens; however, the proteins gave albumen also the characteristics of foaming, emulsifying, and gelling [56]. For some cooking preparations, the quality of albumen is important, and the consumer may evaluate it considering its firmness and consistency around the yolk. Haugh units allow a correct comparison among eggs of different sizes [55]. The Haugh units showed a higher value in PO than in PA. The origin of the breeds seemed to affect the albumen properties also for the tinted eggs, as the Haugh units of ER and PP were higher than those of RM and RL, and also than those of the white eggshell breeds. Even if the daily egg mass may be involved with this trait, other factors may affect the production of the thick albumen, and further research is needed.

The yolk colour is another internal quality trait evaluated by the consumer. This evaluation often does not consider the yolk pigmenting properties in cooking preparations, only the healthiness which a not pale colour may suggest. The yolk colour differed among white eggshell breeds, whereas no differences were detected for the tinted eggshell breeds. PA-C breed showed a Roche scale value lower than PO-B and ER. The reason may be higher egg production and, thus, a dilution effect of the pigments. Also, differences in yolk composition and the vitelline membrane characteristics may have affected the perception of the colour.

## 5. Conclusions

The results indicate that the laying rate changed according to breed, and eggs produced by the eight local breeds of the Veneto region differed in size and many eggshell traits. The consumer may consider not only the exterior aspect of the egg but also the interior quality, as the studied breeds showed differences, especially in the weight of albumen and yolk, the ratio between them, the meat and blood inclusions, and the Haugh units. Further studies are needed for these breeds reared outdoors, to investigate changes in the egg quality according to the age of the hens and the environmental conditions. Many of the studied egg traits allow the consumer to distinguish the eggs for their genetic origin through the eggshell shape and colour, and to use these table eggs for different purposes. For each breed, an egg quality profile should be drawn up and referred to the different stages of the laying cycle, with the purpose to indicate, on the egg carton, the eggshell, albumen, and yolk traits.

## Figures and Tables

**Table 1:** Origin and main characteristics of hens of local chicken breeds [13,24].

Breed	Origin	Comb	Plumage	Adult Body Weight	Productive Attitude
**White eggshell**				
Padovana	XIV century From Polish and other tufted breeds	Absent. Presence of tuft and cranial hernia	Chamois, Golden, and other	1.5–2.1 kg	Egg
Polverara	XV century From Padovana breed	Absent. Presence of tuft	White, Black	1.3–1.8 kg	Egg
**Tinted eggshell**					
Pepoi	XIX century	Single	Golden	1.0–1.3 kg	Dual purpose
Ermellinata di Rovigo	XX century From Sussex and Rhode Island breeds	Single	White plumage with black feathers of hackle and tail	2.2–2.6 kg	Dual purpose
Robusta Maculata	XX century From Brown Orpington and White America breeds	Single	White plumage with feathers with black ends, black feathers of tail	2.8–3.3 kg	Dual purpose
Robusta Lionata	XX century From Brown Orpington and White America breeds	Single	Brown plumage with black feathers of tail and wings	2.8–3.3 kg	Dual purpose

**Table 2:** Least squares means of eggshell traits of the breeds.

	PA-C	PA-G	PO-W	PO-B	PP	ER	RM	RL	RMSE
Egg width, cm	4.21 ^b^	4.18 ^b^	4.09 ^c^	4.04 ^c^	4.04 ^c^	4.25 ^b^	4.35 ^a^	4.33 ^a^	0.137
Egg length, cm	5.40 ^c^	5.61 ^b^	5.37 ^cd^	5.48 ^c^	5.28 ^d^	5.81 ^a^	5.70 ^ab^	5.78 ^a^	0.204
Shape index, %	77.9 ^a^	74.6 ^cd^	76.2 ^abc^	73.8 ^d^	76.6 ^ab^	73.2 ^d^	76.4 ^abc^	74.9 ^bcd^	3.268
Area-to-volume ratio	1.31 ^b^	1.31 ^b^	1.33 ^a^	1.34 ^a^	1.36 ^a^	1.28 ^c^	1.26 ^c^	1.26 ^c^	0.047
L	90.7 ^b^	92.7 ^a^	91.2 ^ab^	90.3 ^b^	84.9 ^c^	77.1 ^e^	67.6 ^f^	80.8 ^d^	3.277
a*	−0.17 ^e^	−0.61 ^e^	−0.22 ^e^	−0.29 ^e^	2.80 ^d^	7.97 ^b^	11.6 ^a^	4.68 ^c^	1.913
b*	5.89 ^e^	3.88 ^f^	6.87 ^e^	6.46 ^e^	14.7 ^d^	21.1 ^b^	24.2 ^a^	17.1 ^c^	3.204
Eggshell thickness ^2^, µm	348 ^b^	355 ^ab^	368 ^a^	356 ^ab^	340 ^bc^	321 ^c^	374 ^a^	336 ^bc^	27
Eggshell weight ^3^, g	4.96 ^c^	5.14 ^bc^	5.20 ^bc^	5.17 ^bc^	4.80 ^c^	4.88 ^c^	5.77 ^a^	5.41 ^ab^	0.49
Eggshell ratio ^3^, %	9.41 ^c^	9.11 ^c^	10.3 ^a^	10.3 ^a^	10.0 ^ab^	8.44 ^d^	9.59 ^bc^	9.12 ^c^	0.65

PA-C: Padovana Camosciata; PA-G: Padovana Dorata; PO-W: Polverara Bianca; PO-B: Polverara Nera; PP: Pepoi; ER: Ermellinata di Rovigo; RM: Robusta Maculata; RL: Robusta Lionata. RMSE: root mean square error. Different letters within traits indicate statistically different means (a,b,c,d,e,f: *p* < 0.05). * part of the definition of a* and b*, as indicated in M&M. Observations (*n*) = 60 per breed. ^2^ Observations (*n*) = 35 per breed. ^3^ Observations (*n*) = 25 per breed.

**Table 3:** Least squares means of albumen and yolk traits of the breeds.

	PA-C	PA-G	PO-W	PO-B	PP	ER	RM	RL	RMSE
**Albumen**									
Weight, g	30.5 ^bc^	32.0 ^b^	28.8 ^cd^	27.9 ^d^	28.7 ^cd^	35.1 ^a^	35.0 ^a^	36.6 ^a^	2.371
Ratio, %	58.0 ^c^	56.8 ^cd^	57.4 ^c^	55.9 ^d^	59.8 ^b^	60.8 ^ab^	58.1 ^c^	61.5 ^a^	1.670
HU ^2^	94.6 ^b^	89.7 ^c^	94.8 ^b^	94.9 ^b^	97.7 ^ab^	100.2 ^a^	94.2 ^bc^	94.0 ^bc^	4.788
**Yolk**									
Weight, g	17.2 ^bc^	19.2 ^a^	16.2 ^c^	16.8 ^bc^	14.5 ^d^	17.8 ^b^	19.4 ^a^	17.4 ^bc^	1.358
Ratio, %	32.6 ^bc^	34.0 ^a^	32.3 ^c^	33.7 ^ab^	30.2 ^d^	30.8 ^d^	32.2 ^c^	29.4 ^d^	1.625
**Egg**									
Yolk-to-albumen ratio	0.56 ^bc^	0.60 ^ab^	0.56 ^bc^	0.61 ^a^	0.51 ^d^	0.51 ^d^	0.56 ^c^	0.48 ^d^	0.042

PA-C: Padovana Camosciata; PA-G: Padovana Dorata; PO-W: Polverara Bianca; PO-B: Polverara Nera; PP: Pepoi; ER: Ermellinata di Rovigo; RM: Robusta Maculata; RL: Robusta Lionata. RMSE: root mean square error. Different letters within traits indicate statistically different means (a,b,c,d: *p* < 0.05). Observations (*n*) = 25 per breed. ^2^ Observations (*n*) = PA-C, PA-G, PO-W, PO-B (22), PP (22), ER (22), RM (21), RL (20).

## Data Availability

The data presented in this study are available on reasonable request from the corresponding author. The data are not publicly available due to their sensitive nature.
